# New developments and future opportunities in biomarkers for amyotrophic lateral sclerosis

**DOI:** 10.1186/s40035-015-0040-2

**Published:** 2015-09-30

**Authors:** Xueping Chen, Hui-Fang Shang

**Affiliations:** Department of Neurology, West China Hospital, Sichuan University, 610041 Chengdu, Sichuan China

## Abstract

Modern technology has improved the ability to probe effectively the underlying biology of ALS by examination of genomic, proteomic and physiological changes in patients with ALS, as well as to monitor functional and structural changes during the course of disease. While effective treatments for ALS are lacking, the discovery of sensitive biomarkers to disease activity offers clinicians tools for rapid diagnosis and insights into the pathophysiology of ALS. The ultimate aim is to lessen reliance on clinical measures and survival as trial endpoints and broaden the therapeutic options for patients with this disease.

## Introduction

The earliest descriptions of amyotrophic lateral sclerosis (ALS) were made in the late 1800s, and the understanding of the clinical and pathological heterogeneity of ALS has made a major advance in the last 30 years. However, the phenotypic variability and a common clinical syndrome, including the region of onset, rate of progression, patterns of disease spread, and relative burden of upper motor neuron (UMN), lower motor neuron (LMN), and cognitive pathology complicates the diagnosis of disease and the measurement of disease progression. For example, the mean delay in time from presentation of symptoms to diagnosis has remained at over 1 year [1], and around 5 ~ 10 % of patients with ALS survive for more than a decade after diagnosis [2, 3]. Furthermore, nearly one-quarter of patients who present with progressive muscular atrophy (PMA) develop signs of UMN disease within 5 years of diagnosis [4]. while patients with primary lateral sclerosis (PLS) presenting isolated UMN signs evolve LMN features over time [5]. The presence of UMN-predominant and LMN-predominant clinical signs is associated with better prognosis [3, 6]; although within these phenotypes there may still be dramatic variability in the rate of disease progression. All the heterogeneity of clinical presentation indicates that quantitative monitoring biomarkers which would facilitate effective decision-making and care-planning are the most needed. Technological advancements have led to the discovery of candidate biomarkers for ALS in biofluids and tissues, electrophysiological indicators, and neuroimaging measures. This review highlights advances in the identification and understanding of biomarkers of ALS, including biofluid and tissue biomarkers, neurophysiology biomarkers, and neuroimaging biomarkers.

### Tissue and biofluid biomarkers (Table 1)

**Table 1:** Candidate-tissue and biofluid-based biomarkers for ALS

	Evaluated biomarkers	Meaning/function
CSF	MMP-2, MMP-9	Markers of BBB dysfunction
	NfL, pNFH/C3, p-tau	Neuroaxonal degeneration markers
	cystatin C, TDP-43	Markers of the neuroprotection
	IL-6, IL-8, complement factors C3 and C4, prostaglandin E2, neopterin, peroxynitrite, G-CSF, MCP-1	Markers of inflammation and immune activation
	S100b, EPO	Glial activation markers
Blood	Creatinine, albumin, ferritin	
Muscle	Nogo-A, smads	

#### Biofluids

##### Cerebrospinal fluid (CSF)

CSF is an ideal biofluid for biomarker discovery due to its approximation to the brain and spinal cord regions. It might reflect pathophysiological alterations in disease progression, and it could provide an insight into disease pathogenesis.

### Dysfunction of the blood brain barrier (BBB) and its markers in CSF

The changes in the selected matrix metalloproteinases (MMPs), including MMP-2 and MMP-9, were demonstrated in the CSF of ALS patients. One study showed that the concentrations of MMP-2 were higher, and the MMP-9 concentrations were lower in the CSF of ALS patients than in healthy controls [7]. However, another study presented an opposite findings and they found that the CSF MMP-9 concentrations in ALS patients were significantly higher than in healthy controls, and there were no significant differences of CSF MMP-2 concentrations between ALS patients and controls [8]. MMPs are involved in mediation of disruption of BBB, and contribute to ALS pathology, but future researches concerning the significance of selected MMPs as potential biomarkers for ALS need to be continued.

### Neuroaxonal degeneration markers in CSF

Neurofilaments (Nf) are considered to be an important component of the axonal skeleton. Nf are composed of three subunits: a light (NfL), a medium (NfM) and a heavy (NfH) chain. Tortelli et al. reported that the concentrations of NfL in CSF were significantly higher in ALS cases than in neurological controls [9]. They also showed that CSF NfL levels was correlated with the time of symptom spreading from spinal or bulbar localization to both (TTG), a clinical intermediate parameter of survivorship [10]. In addition, recently, another study demonstrated that the NfL levels in CSF discriminated between ALS patients and neurological controls, with a sensitivity of 97 % and specificity of 95 %. Furthermore, CSF NfL was highly correlated with serum levels, which were found to be strong, independent predictors of survival [11]. The prognostic and diagnostic values of the CSF levels of phosphorylated neurofilament heavy chain and complement C3 (pNFH/C3) were also confirmed in patients with ALS, and the predictive pNFH/C3 ratio identified ALS with 87.3 % sensitivity and 94.6 % specificity [12]. A final prospective clinical qualification study is currently underway using 4 sites in the US and 2 sites in Europe. The CSF Nf levels could be promising disease-monitoring biomarkers in ALS targeting cytoskeletal antigens. Another biomarker of axonal degeneration is Tau protein. Significantly reduced CSF levels of p-tau and the p-tau:t-tau ratio were identified in ALS [13]. However, contrary to this report, another study showed that CSF p-tau was not significantly reduced, and t-tau was significantly increased in ALS cases [14]. Thus, it is still controversial to consider CSF p-tau as a suitable diagnostic biomarker for ALS.

### Markers of the neuroprotection in CSF

Cystatin C, a cysteine proteinase inhibitor, has been implicated in the processes of neuronal degeneration and the repairmen of the nervous system [15], and a significant decrease in cystatin C in CSF of ALS patients has been described in previous studies [16–20]. However, another study reported that there was no difference in CSF cystatin C levels in patients and controls from six European centers [21]. These inconsistent findings may be explained partially by the fact that cystatin C seems to be prone to preanalytic artifacts [22]. TDP-43 (43-kDa transactive response (TAR)-DNAbinding protein), regulating biological processes in the nucleus, plays a crucial role in the neurodegeneration in ALS. TDP-43 was considered to be a biomarker for the early stages of disease, since significantly higher levels of CSF TDP-43 were identified in ALS patients at the disease onset [23]. Similar findings also showed that CSF TDP-43 levels were increased only in ALS patients with a sensitivity of 59.3 % and a specificity of 96.0 %, and the lower CSF TDP-43 levels may be associated with shorter survival time [24]. However, another study concludes that TDP-43 in CSF originates mainly from blood, and measurements of TDP-43 in CSF and blood may be of minor importance as a diagnostic tool [25].

### Markers of inflammation and immune activation in CSF

Inflammation in the CNS and the systemic circulation is considered to be a key factor in the pathogenesis of ALS [26, 27]. Inflammation in ALS is resulted from activation of microglia and autoimmune responses in the CNS, leading to neuronal dysfunction. In the CSF, increased concentrations of interleukin-6 (IL-6), interleukin-8 (IL-8), complement factors C3 and C4, prostaglandin E2, neopterin, peroxynitrite, granulocyte colony stimulating factor (G-CSF), monocyte chemoattractant protein-1 (MCP-1), and antibodies against various cellular structures have been identified [28–30]. CHIT-1 is a enzyme synthesized by microglia or infiltrating macrophages [31]. Studies showed that CSF CHIT-1 levels were significantly higher in SALS patients than in other neurological controls [32, 33]. The enhanced expression of CHIT-1 possibly indicates a neuroinflammatory response activated by microglia, and an index of the severity of inflammation alongside the release of pro-inflammatory cytokines [34]. Thus, CHIT-1 may be helpful for the evaluation of cerebral inflammatory activity in ALS patients.

### Glial activation markers in CSF

Glial activation occurs in early stages of during the cascade of neuroaxonal degeneration [35]. Markers of glial activation include erythropoietin (EPO), S100 beta (S100b), glial fibrillary acidic protein (GFAP) and glutamine synthetase. In ALS, CSF concentrations of EPO were significantly decreased [36, 37]. CSF S100b levels were found to be significantly lower in lower MND (LMND), as compared to other MNDbut there is no consistent evidence for a correlation between concentrations of CSF S100b and disease severity. . Furthermore, changes of S100b have been reported in other neurodegenerative diseases. Therefore, S100b has limited usefulness for disease diagnosing and monitoring disease progression [38].

### Blood

Blood is more accessible compared to CSF. In ALS, serum albumin and creatinine are reliable markers of the severity of clinical status and can be used in defining prognosis at the time of diagnosis [39]. The DREAM-Phil Bowen ALS Prediction Prize4Life challenge also confirmed that uric acid and creatinine could be used as potential nonstandard predictors of disease progression, shedding light on ALS pathobiology [40]. A post hoc analysis of subgroup outcomes and creatinine in the phase III clinical trial (EMPOWER) of dexpramipexole in ALS demonstrated that creatinine loss correlated with disease progression [41]. Similar findings also showed that changes to ferritin and creatinine levels with time were associated with ALS progression, suggesting serum creatinine as a candidate biomarker [42].

### Muscle

Skeletal muscle is one of the most severely affected by the disease and it is easily accessible to biopsy. Thus, with progressive denervation and atrophy, muscles may represent a valuable source of biomarkers in ALS. Nogo-A was found to be strongly expressed in ALS muscles, and its expression was correlated with amyotrophic lateral sclerosis functional rating scale (ALSFRS) [43, 44]. However, studies questioned that expression of Nogo-A in human muscle fibers may be not specific for ALS [45, 46]. Muscle transcriptome analyses have found that smad1, 5, 8 mRNA and protein levels, as well as Smad phosphorylation, were elevated in ALS muscle. Therefore, muscle smads could serve as potential candidates for ALS biomarkers [47].

### Physiological biomarkers (Table 2)

**Table 2:** Candidate physiological biomarkers for ALS

	Evaluated biomarkers
Measures of LMN loss	Motor unit number estimation (MUNE)
	Axonal excitability
	Electrical impedance myography (EIM)
	Muscle ultrasound (MUS)
Measures of UMN loss	Transcranial magnetic stimulation (TMS)

While biochemical markers may provide clues for the specific cellular or signaling alterations that occur in ALS, a number of global physiological features can be assessed that might differentiate ALS from other neurological diseases and enable the monitoring of disease progression. The presence of fibrillation potentials and positive sharp waves on needle electromyography indicates ongoing LMN degeneration or axonal loss, and prolonged and polyphasic motor units are considered to be a consequence of reinnervation. However, electromyography has a limited sensitivity (60 %) for the diagnosis of ALS, and the characteristics measured, including motor unit duration, amplitude and phase do not systematically change with disease progression. A measure of motor unit loss that is reproducible, noninvasive, rapidly obtained, and amenable to repeated evaluation over time would be highly desirable.

### Measures of LMN Loss

#### Motor unit number estimation (MUNE)

Motor unit number estimation (MUNE) is a neurophysiological tool that was developed to quantify residual motor axons supplying a muscle, by estimating the contribution of individual motor units to the maximum response amplitude. Longitudinal studies of changes in MUNE in ALS have correlated loss of motor neurons with survival [48]. A number of MUNE techniques for estimating the average amplitude of single motor units have been developed, but most of them have been limited by sampling bias and lack of reproducibility [49]. Recently, multipoint incremental MUNE was found to have excellent test-retest reliability. The rate of decline was more sensitive than that of MRC sum score and ALSFRS-R [50, 51]. Other new MUNE methods, including Bayseian MUNE and motor unit number index (MUNIX), the latter was considered to be a reliable electrophysiological biomarker to track lower motor neuron loss in ALS [52]. Bayesian MUNE could be used to show differing rate of loss of motor units in subgroups of ALS [53].

### Axonal excitability

Motor axonal dysfunction has been demonstrated in ALS patients using threshold-tracking technology, with increased persistent conduction in sodium channels and reduced conduction in potassium channels [54, 55]. Changes in axonal excitability evolve with disease progression [56], and may be used as a predictor of survival in ALS patients [57]. Axonal excitability parameters could be used as biomarkers of axonal degeneration.

### Electrical impedance myography (EIM)

EIM is an emerging technology in which a high-frequency, low-intensity electrical current is applied to a localized area of muscle and the consequent surface voltages measured [58]. EIM assesses the integrity and structure of the muscle. Recently, a multicenter study compared EIM directly to the ALSFRS-R, MUNE, and handheld dynamometry, and found that EIM outperformed the other measures in terms of its ability to detect deterioration [59], and EIM can serve as a meaningful measure of disease severity in ALS [60]. One advantage of EIM is its ability to assess a variety of muscles and to measure specifically the area of the body where the disease is progressing most rapidly. The other advantage is that EIM of tongue musculature could distinguish patients with ALS from healthy controls. The demonstrated relationship between tongue function and Ph supports further testing of EIM of the tongue as a potential biomarker in ALS [61]. Large studies are needed to further refine the technique for easy use and validate it as a biomarker for future clinical trials.

### Muscle ultrasound (MUS)

Ultrasound may also detect changes in the thickness and echogenicity in muscles with and without clinical weakness [62]. Muscle ultrasound differentiated between ALS and mimics with 96 % sensitivity and 84 % specificity, and it is a sensitive tool to screen for regional lower motor neuron involvement [63]. The most established role of MUS in the ALS clinic relates is the identification of fasciculations. The sensitivity and specificity of MUS in diagnosing ALS was almost equivalent to those of EMG, and combined use of EMG and MUS enhanced the diagnostic accuracy compared to EMG alone [64].

### Measures of UMN Loss

#### Transcranial magnetic stimulation (TMS)

TMS is a neurophysiological test that measures UMN functional integrity, and it is able to improve the sensitivity of ALS diagnosis by demonstrating evidence of subtle subclinical UMN dysfunction, as well as clarify the relationship between ALS and its variants [65], such as PMA. It is used to study the excitability and conductivity of the corticospinal system. Changes in cortical excitability may precede the development of muscle weakness in ALS [66, 67]. Single pulse evoked TMS amplitude could used to objectively discriminate ALS from neurological controls and assess the progression of ALS [68–70]. The threshold tracking TMS technique could used to differentiate ALS from non-ALS disorders with a sensitivity of 73.21 % and specificity of 80.88 % at an early stage in the disease. It may represent a useful diagnostic investigation to prove UMN dysfunction at early stages of ALS when combined with the Awaji criteria [71].

### Neuroimaging biomarkers (Table 3)

**Table 3:** Candidate neuroimaging biomarkers for ALS

	Evaluated biomarkers
Radionuclide imaging	SPECT
	PET
Magnetic resonance imaging (MRI)	Voxel & surface-based MRI morphometry (VBM&SBM)
	Diffusion tensor imaging (DTI)
	Functional MRI (fMRI)
	Magnetic resonance spectroscopy (MRS)
	Spinal cord MRI

Imaging offers a noninvasive approach to biomarker discovery and disease monitoring. If neuroimaging biomarkers were validated, they could be easily integrated into routine clinical evaluation of patients with suspected ALS, and revealed disease mechanisms that might aid the discovery of novel drug targets.

### Radionuclide imaging

Single photon emission computed tomography (SPECT) is a practical and potentially widely applicable form of radionuclide imaging. It was at the forefront of the now established concept of a continuum between ALS and frontotemporal dementia (FTD) [72]. Positron emission tomography (PET) has greater resolution than SPECT. Pivotal ‘activation’ PET studies, using tracers sensitive to blood flow and metabolism, provide in vivo evidence for a consistent extramotor cerebral pathology in ALS [73], while ‘ligand’ PET is used to identify specific cerebral neuronal receptor changes in ALS. The PET ligand 11C-PK11195 binds to the peripheral benzodiazepine receptor, which are expressed by activated microglia. A study provided *in vivo* evidence of widespread corticospinal tract and extra-motor microglial activation in ALS patients [74]. A serotonin 5-HT1A receptor PET ligand 11C-WAY100635 showed marked reductions in binding in a group of nondepressed ALS patients [75]. Loss of binding was mainly located in frontotemporal regions. These locations are similar in distribution to a subsequent study in patients with FTD [76], and this striking reduction in serotonin-1A receptor binding was confirmed histologically [77]. The future value of PET in ALS will depend on the development of ligands with relevance to pathogenic hypotheses, e.g., more specific neuroinflammatory or protein markers.

### Magnetic resonance imaging (MRI)

The observation of corticospinal tract hyperintensity lacks sensitivity and specificity for the diagnosis of ALS. Routine clinical MRI has limited value as a source of biomarkers in ALS, e.g., the marked precentral gyrus atrophy was demonstrated in rare cases of PLS. Thus, the advanced analysis methods have greater potential in this regard.

### Voxel & surface-based MRI morphometry

Automated and unbiased whole-brain analysis techniques have been developed to quantify and segment grey and white matter (WM) morphology using T1-weighted images, and this advanced analysis techniques include voxel- and surface-based morphometry (VBM and SBM), the latter is known as cortical thickness measures, because it allows decomposition of cortical volume into both thickness and surface area and respects the cortical topology, with enhanced reliability and sensitivity [78]. A meta-analysis of VBM studies in ALS demonstrated significant grey matter loss in the right precentral gyrus [79]; however, extra-motor changes were not found to be a consistent feature. This finding is in agreement with clinical observations, since at least fifty percent ALS patients have no detectable cognitive impairment [80, 81]. However, in those ALS patients with significant cognitive impairment or frank dementia, extra-motor grey matter changes are considered to be a clear feature across a range of techniques [82]. Two longitudinal VBM studies have showed progressive atrophy in extra-motor as well as motor regions [83, 84], and patients with more rapidly progressive ALS presented frontal lobe changes as well as more extensive motor changes [84]. This separate observations reflect that early cognitive impairment is a poor prognostic factor [85]. Studies of SBM in ALS have demonstrated cortical thinning in the precentral gyrus [86–88], and a marked correlation was found between thinning within the temporal lobe cortex and rapid disease progression [88].

### Diffusion tensor imaging

Post mortem histopathological study have demonstrate widespread cerebral white matter tract damage in ALS, and this alteration can now be detected non-invasively using diffusion tensor imaging (DTI) [89]. The two main quantitative measures of loss of neuronal tract integrity are increased mean diffusivity (MD) and reduced fractional anisotropy (FA). DTI studies have shown consistently reduced FA in the corticospinal tract (CST) and corpus callosum of ALS patients, particularly within the posterior limb of the internal capsule (PLIC) [90, 91]. Targeted FA measurement at the PLIC may provide prognostic information [92]. Many DTI studies showed that decreased CST FA in ALS patients was correlated with disease severity and rate of disease progression, along with clinical and electrophysiological measures of UMN degeneration, but paradoxically higher CST FA values were reported in two studies. Increased MD of the CST was associated with longer disease duration [93]. A meta-analysis has demonstrated the independent prognostic value of CST FA [90]. DTI studies that employed a voxel-wise approach also demonstrated a decrease of FA values in regions outside the ‘classical’ motor network [93, 94].

### Functional MRI

Blood oxygenation level-dependent (BOLD) functional MRI (fMRI) studies of motor tasks in ALS patients is unique in its ability to study cerebral activity noninvasively, confirming the widened region of activation observed in PET studies. More recently, it is the study of the ‘resting state’ that shows multisystem involvement of cognitive, emotional and sensory processing pathways in ALS, suggesting novel insight into ALS as a ‘system failure’. Resting-state fMRI (R-fMRI) has shown increased functional connectivity within the damaged ALS cortical network, with possible implications in relation to cortical inhibitory influences [95, 96]. The combination of structural and functional MRI measures holds major promise for more sensitive biomarker panels in ALS, providing much better separation of ALS phenotypes from healthy age-matched controls [95].

### Magnetic resonance spectroscopy

Magnetic resonance spectroscopy (MRS) is an application of MRI that permits the noninvasive quantification of cerebral tissue metabolites. *N*-acetylaspartate (NAA), total creatine (Cr) and total choline (Cho) have been mostly studied due to their simple (singlet) spectral patterns and relatively high concentrations in the CNS. It has consistently demonstrated reduced NAA ratios (a non-specific marker of neuronal loss) in the motor cortex of ALS patients, and high-field studies also suggest a specific loss of GABA-ergic influence [97]. Metabolite changes have also been found in the brainstem of ALS patients [98, 99]. MRS studies have found decreased NAA:Cho in nonmotor regions, including the thalamus and basal ganglia [100], mid-cingulate cortex [101] and the frontal and parietal lobes [102] of patients with ALS.Longitudinal MRS studies have reported continued reduction in NAA:Cho and NAA in the primary motor cortex (PMC) of ALS patients during follow-up periods, with a correlation between changes in PMC NAA:Cho and progression rate [98].

### Spinal cord MRI

The ‘dying-back’ theory of ALS suggests that early degeneration is more likely to be captured at the spinal anterior horn rather than the brain [103]. Patients with ALS have been shown to have increased radial diffusivity and considerably reduced FA in the spinal cord, particularly in the distal cervical cord. Particularly, FA values correlated with disability, and focal atrophy of the spinal cord correlated with muscle deficits [104, 105]. Longitudinal reductions in cord FA and elevation in cord MD have been shown in ALS patients after a mean follow-up of 9 months [106]. Reduced NAA:Cr and NAA:myo-inositol ratios have been reported in the cervical cord in ALS patients [107, 108] and in presymptomatic carriers of *SOD1* mutations [109], raising the potential for screening and early detection.

## Conclusions

Multiple methodological advancements have led to the discovery of various biomarkers for ALS. Importantly, many biomarkers are emerging with the potential to refine the diagnosis, stratify patients prognostically, and facilitate therapeutic development. They also have provided mechanistic insights, since it seems likely that there are multiple, possibly more discrete, pathways converging on motor neuron degeneration. Each class of biomarker requires continued development. A key aim for further biomarker development is the combination of different classes to create a ‘signature’ applicable to the range of phenotypes, and which can provide quantifiable evidence of efficacy in future therapeutic trials and successful translation to the clinical setting. Future longitudinal study with a large group of patients in several disease phenotypes will be required to validate a panel of biomarkers that could be easily incorporated into the routine clinical evaluation of patients with suspected ALS. Combination of measurements in biofluids or tissues with advanced technologies in neurophysiology and neuroimaging will increase sensitivity and accuracy for acute diagnosis and analyzing ALS disease progression and prognosis.
